# Adiponectin Enhances the Responsiveness of the Olfactory System

**DOI:** 10.1371/journal.pone.0075716

**Published:** 2013-10-10

**Authors:** Diana Loch, Christian Heidel, Heinz Breer, Jörg Strotmann

**Affiliations:** Institute of Physiology, University of Hohenheim, Stuttgart, Germany; Monell Chemical Senses Center, United States of America

## Abstract

The peptide hormone adiponectin is secreted by adipose tissue and the circulating concentration is reversely correlated with body fat mass; it is considered as starvation signal. The observation that mature sensory neurons of the main olfactory epithelium express the adiponectin receptor 1 has led to the concept that adiponectin may affect the responsiveness of the olfactory system. In fact, electroolfactogram recordings from olfactory epithelium incubated with exogenous adiponectin resulted in large amplitudes upon odor stimulation. To determine whether the responsiveness of the olfactory sensory neurons was enhanced, we have monitored the odorant-induced expression of the immediate early gene *Egr1*. It was found that in an olfactory epithelium incubated with nasally applied adiponectin the number of *Egr1* positive cells was significantly higher compared to controls, suggesting that adiponectin rendered the olfactory neurons more responsive to an odorant stimulus. To analyze whether the augmented responsiveness of sensory neurons was strong enough to elicit a higher neuronal activity in the olfactory bulb, the number of activated periglomerular cells of a distinct glomerulus was determined by monitoring the stimulus-induced expression of *c-fos*. The studies were performed using the transgenic mOR256-17-IRES-tauGFP mice which allowed to visualize the corresponding glomerulus and to stimulate with a known ligand. The data indicate that upon exposure to 2,3-hexanedione in adiponectin-treated mice the number of activated periglomerular neurons was significantly increased compared to controls. The results of this study indicate that adiponectin increases the responsiveness of the olfactory system, probably due to a higher responsiveness of olfactory sensory neurons.

## Introduction

The necessity to find suitable food sources is desperately needed when the level of internal energy resources, the body fat mass, is running low. The extent of body fat mass is reflected and regulated by two adipokine hormones, adiponectin and leptin, which are considered as long-term modulators of energy balance [1]–[3]. As a consequence of scarce fat reserve the level of circulating adiponectin is high whereas leptin is low [2], [4]. Consequently, adiponectin is considered as a starvation signal which stimulates food intake and decreases energy expenditure [5] and thus contributes to fat accumulation in response to depletion [6]. The effect is supposed to be mediated by neurons in hypothalamic nuclei which express receptors for adiponectin and respond to adiponectin with a change in their electrical excitability [7], [8]. These neurons are supposed to govern the activities which correlate with hunger and satiety, including the control of food intake and the search for food sources. In most mammalian species olfaction plays a critical role in finding suitable food sources and it would be highly beneficial if under conditions of scarce fat reserves the sensitivity of the olfactory system was increased. So one could imagine that an increased olfactory sensitivity induced by starvation signals would increase the probability to find hitherto undetected food sources and thereby the chance for survival. Interestingly, in a recent study it has been found that all mature olfactory sensory neurons (OSNs) in the olfactory epithelium express one of the adiponectin receptor subtypes, namely adiponectin receptor 1 (adipoR1) [9]. The function of adipoR1 receptors in the main olfactory epithelium is still elusive, however, the notion that a starvation signal mediated by adiponectin may be most relevant for OSNs involved in food finding was supported by the observation that the receptor adipoR1 was only expressed in neurons of the main olfactory epithelium and the septal organ but not in neurons of the vomeronasal organ, which are specialized in detecting pheromones and thereby involved in mediating social behavior rather than in food finding [9]. Moreover, it has recently been shown that a polymorphism in the gene encoding AdipoR1 affects olfactory recognition in humans [10]. In this study, attempts were made to explore if adiponectin might in fact lead to changes in the responsiveness of the olfactory sensory system. The results indicate that pretreatment with adiponectin resulted in a higher number of OSNs responding to an odorant and a higher neuronal activity in the olfactory bulb corresponding glomeruli.

## Materials and Methods

### Animals and tissue preparation

For this study, adult wildtype C57BL/6J mice purchased from Charles River (Sulzfeld, Germany) and animals from our previously generated transgenic mouse line which carry a targeted mutation of IRES-tauGFP at the mOR256-17-locus [11] were used. All animals were kept at a 12h/12h light/dark cycle and had free access to water. All animals were fed with standard laboratory chow *ad libitum.* For tissue preparations, animals were killed by cervical dislocation or by CO_2_ asphyxiation and subsequent decapitation.

### Ethics statement

The institutional and national guidelines for the care and use of laboratory animals according to the Society of Laboratory Animals (GV-SOLAS) were followed. The work was approved by the Committee on the Ethics of Animal Experiments at the Regierungspräsidium Stuttgart (#35-9185.81/0296 V260/09Phy) and the University of Hohenheim Animal Welfare Officer (T42/10 Phy).

### Application of adiponectin to the nose

Lyophilized recombinant globular adiponectin was purchased from Phoenix Pharmaceuticals (Burlingame, CA) and resolved in 5 mM Tris, pH 7.6; 10 µl aliquots were frozen at –20°C. For application to the nose, adiponectin was diluted in 5 mM Tris, pH 7.6 to a final concentration of 1 nM which equals a concentration of 16 µg/ml. This concentration is slightly higher than the adiponectin blood concentration of ad libitum fed mice [12]–[15]. 1 µl of the solution was applied to the region of the nostrils. Control animals received buffer solution.

### Exposure to odorants for analysis of *Egr1* expression in the olfactory epithelium

The odorants benzaldehyde, 1-heptanal and 2,3-hexanedione were purchased from Sigma-Aldrich. Before exposure to odorants, mice were placed individually into a closed plastic box (approx. 11.5 cm height×14 cm width×29 cm length) for 60 minutes (min) to reduce expression of *Egr1* due to home cage odors. The floor of the box was covered with cat litter (EAN 4311501304792) to absorb their excretions. After the adiponectin or buffer treatment, mice were placed back into the box for 30 min. Subsequently, they were transferred for 60 min to a second box which contained a filter paper (approx. 2 cm height×4 cm length) with either 10 µl of pure 1-heptanal, 100 µl of benzaldehyde (diluted 1∶100 in water), or 100 µl 2,3-hexanedione (diluted 1∶100 in water). After this time they were sacrificed and dissected for *in situ* hybridisation experiments.

### Exposure to odorant for analysis of *c-fos* expression in the olfactory bulb

For exposure to the odorant 2,3-hexanedione, mice were placed individually into a closed plastic box (approx. 11.5 cm height×14 cm width×29 cm length); the floor was covered with cat litter (EAN 4311501304792) to absorb their excretions. The box had two connections to attach plastic tubes (10 cm length, 0.8 cm inner diameter) on one side, and small holes for the outgoing air on the opposite side. The box was supplied with a constant stream of air (4 L/min) flowing through a charcoal filter and a manual 3-way valve. The valve was adjusted to deliver either a stream of air through tube #1, or air containing an odorant through tube #2. Prior to the application of adiponectin or buffer to the nose and the subsequent odorant exposure, mice were exposed to clean air for 120 min to decrease *c-fos* expression due to home cage odors. 30 min after the application of adiponectin, the odorant (100 µl of 2,3-hexanedione diluted 1∶100 in water) was given onto a piece of filter paper (approx. 2 cm height×4 cm length) and placed into tube #2. Charcoal-filtered air flowing over this material was passed into the box. In order to minimize adaptation of mice to the odorant, it was presented in intervals of 2 min, alternating with pure air for 3 min. This protocol was repeated for 30 min. 60 min after this stimulation period, the mice were sacrificed for *c-fos* analyses.

### *In situ* hybridization

Digoxigenin-labelled antisense riboprobes were generated from partial cDNA clones encoding *c-fos* (NCBI accession number NM_010234) or *Egr1* (NCBI accession number NM_007913.5, position 589 - 1871) in pGEM-T plasmids by using the SP6/T7 RNA labelling system (Roche Diagnostics, Mannheim, Germany), as recommended by the manufacturer. The specificity of the signals was confirmed by control experiments with corresponding sense probes. For sectioning, all bones of the head surrounding the olfactory bulb and the nasal turbinates were removed, freshly prepared tissue was embedded in Tissue Freezing Medium (Leica Microsystems, Bensheim, Germany) and frozen using liquid nitrogen. Cryosections (12 µm) were generated using a CM3050S cryostat (Leica Microsystems) and mounted onto Starfrost microscope slides (Knittel Glass, Braunschweig, Germany). Sections were fixed with 4% paraformaldehyde in 0.1 M NaHCO_3_, pH 9.5 for 45 min at 4°C. Slices were washed in 1 x phosphate-buffered saline (PBS; 0.85% NaCl; 1.4 mM KH_2_PO_4_ and 8 mM Na_2_HPO_4_, pH 7.4) for 1 min, incubated in 0.2 M HCl for 10 min, in 1% Triton X-100 for 2 min, and washed again twice in 1 x PBS for 30 seconds, all at room temperature. Finally, sections were incubated in 50% formamide/5x standard sodium citrate (SSC; 1 x SSC: 0.15 M NaCl; 0.015 M Na-citrate, pH 7.0) for 10 min. The tissue was then covered with hybridization buffer (50% formamide with 2 x SSC, 10% dextran sulphate, 0.2 mg/ml yeast t-RNA and 0.2 mg/ml sonicated herring sperm DNA) containing the probe and incubated in a humid chamber (50% formamide) at 65°C overnight. For posthybridization, slides were washed twice for 30 min in 0.1 x SSC at 65°C, then incubated with 1% blocking reagent (Roche) in Tris-buffered saline (TBS; 100 mM Tris, pH 7.5 and 150 mM NaCl) with 0.3% Triton X-100 for 30 min at room temperature. Afterwards the slices were incubated with an anti-digoxigenin alkaline phosphatase-conjugated antibody (Roche) diluted 1:750 in blocking reagent with 0.3% Triton X-100 for 30 min at 37°C. After two washes in TBS for 10 min, slides were shortly rinsed in alkaline phosphatase (AP) buffer (100 mM Tris, pH 9.5; 100 mM NaCl and 50 mM MgCl_2_). Hybridization signals were visualized using nitroblue tetrazolium (NBT) and 5-bromo-4-chloro-3-indolyl phosphate (BCIP) in AP buffer as substrate. Finally, the sections were rinsed with H_2_O and mounted in MOWIOL (33% glycerine, 13% polyvinylalcohol 4-88 [Sigma] in 0.13 M Tris pH 8.5).

### Immunohistochemistry

For sectioning, all bones of the head surrounding the olfactory bulb and the nasal turbinates were removed. For the immunohistochemical detection of *c-fos* in the olfactory bulb of transgenic mice, the tissue was fixed by immersion in 4% paraformaldehyde (in 150 mM phosphate buffer, pH 7.4) for 15 min on ice, cryoprotected in 25% sucrose overnight at 4°C, embedded in Tissue Freezing Medium and frozen using liquid nitrogen. Cryosections (12 µm) were adhered to Superfrost slides (Menzel, Braunschweig, Germany). Before processing, the sections were air-dried for 30 min and then rinsed in 1 x PBS for 10 min at room temperature. Rabbit anti-*c-fos* antibody (Santa Cruz Biotechnology) was diluted 1:450 in 0.3% Triton X-100 in 1 x PBS containing 10% normal goat serum (NGS; Dianova, Hamburg, Germany). Sections were incubated with the diluted primary antibody overnight at 4°C. After 3 rinses for 5 min in PBS, the bound primary antibody was visualized by incubating the appropriate secondary antibody conjugated to Alexa 488 or Alexa 568 (Invitrogen) diluted in PBS/0.3% Triton X-100 containing 10% NGS for 2 hours at room temperature. After washing for 3 times for 5 min, the sections were counterstained with 4′,6-Diamidin-2′-phenylindoldihydrochlorid (DAPI) (1 µg/ml, Sigma Aldrich) for 3 min at room temperature, rinsed with H_2_O and mounted in MOWIOL.

### Microscopy and photography

Sections were analyzed using a Zeiss Axiophot microscope (Carl Zeiss MicroImaging). Images were captured using a Zeiss Axiocam for transmitted light and a “Sensi-Cam” CCD camera (PCO-imaging) for fluorescent images.

### Quantitative analyses

For cell counts, the sections were examined with a 10x objective for transmitted light preparations and with a 40x objective for fluorescent preparations. For counts of *Egr1*-positive cells in the olfactory epithelium, 50 consecutive sections through ectoturbinate 2 or endoturbinate III, respectively, were analyzed from each individual. The first section was defined as the section on which ectoturbinate 2 became separated. The number of Egr1-positive cells was determined and given as means ± SD. Quantitative analyses of juxtaglomerular cells (JC) in the olfactory bulb were performed according to Bautze et al. [16]. The mOR256-17-glomerulus was defined as the region of GFP-labelled neuropil delimited by DAPI-stained juxtaglomerular cells; medial and lateral glomeruli were taken into account. *c-fos* immunoreactive and DAPI-stained cells that were immediately adjacent to the glomeruli (maximal 4 nuclei widths from the outer boundary of the axon fibers within the glomerulus) were counted on serial sections; *c-fos* signals were counted when their signal intensity was above backround and the signal was colocalized with a DAPI-stained nucleus. The percentage of *c-fos* immunoreactive cells from the DAPI-stained cells surrounding the glomeruli was determined and given as means ± SD. Statistical analyses were performed by using the unpaired *t*-test in GraphPad Prism (GraphPad Software, www.graphpad.com). For statistical evaluation we set P<0.05 as significant, P<0.01 as very significant and P<0.001 as extremely significant.

### EOG recordings

EOG recordings were performed from the medial surface of the nasal turbinates in an opened nasal cavity configuration. For this purpose, the mouse head was cut longitudinally along the midline to expose the endoturbinates. The hemihead was embedded in plastilin and positioned onto a recording stage under a stereomicroscope (Leica MS5, Leica Microsystems, Wetzlar, Germany). As recording and reference electrodes, chlorided silver wires in a pulled borosilicate glass capillary (1B100F-3; World Precision Instruments, Berlin, Germany) filled with modified Ringer's solution (138 mM NaCl, 5 mM KCl, 2 mM CaCl_2_, 1.5 mM MgCl_2_, 10 mM glucose, 10 mM HEPES, pH 7.3) were used. The recording electrode was positioned onto the surface of the epithelium, the reference electrode onto the surface of the brain using manual micromanipulators (M3301R M3301L, World Precision Instruments). Throughout the experiment, the hemi-head was kept under a constant flow of humidified air (50 ml/min) through an exchangeable Pasteur pipette with a 5 mm opening positioned 3 cm distant from the epithelial surface. For odor stimulation, puffs of odorized air (500 ms duration; 3 psi) were generated by a picospritzer-II (Parker Hannifin Corporation, Pine Brook, NJ, USA) and inserted through a y-shaped connector into the constant air flow through. Odorized air was produced by placing 0.2 ml solution of odorant diluted 1∶1000 or 1∶100.000 in water into a 1,5 ml Eppendorf tube equipped with 18-gauge needles as input and output ports. EOG signals were recorded using a Duo773 electrometer (World Precision Instruments) with digital low-pass filter at 20 Hz and digitized at a rate of 2 kHz using a NuDAQ PCI9112 device (Bedo Elektronik, Krefeld, Germany) interfaced to a Pentium PC with DasyLab Software (Geitmann GmbH, Menden, Germany). To test the effect of adiponectin on the responsiveness of the olfactory epithelium, 10 µl adiponectin solution (100 nM in Ringer) was applied to the recording site using glass micropipettes (1 mm diameter). Responses were recorded 5 min prior and 15 min after the application. Control experiments were performed using buffer solution without adiponectin. All results were analyzed and graphed using Origin6.0 (OriginLab Corp., Northampton, MA, USA).

## Results

**Figure 1:**
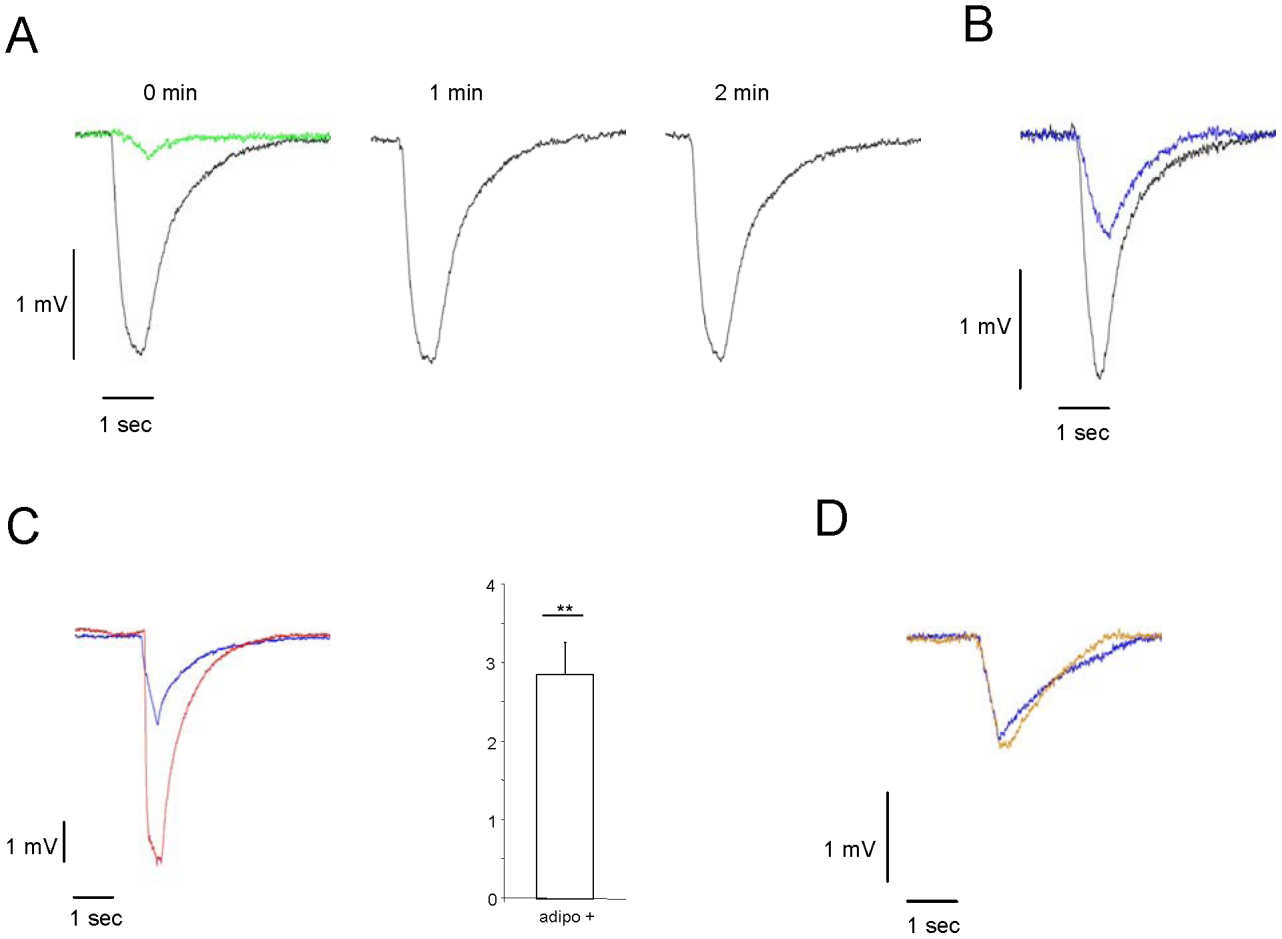
Adiponectin increases the electrical response of olfactory sensory neurons to benzaldehyde. (**A**) Representative EOG responses to benzaldehyde (1:1000) (black traces) recorded from one position on the medial surface of endoturbinate II at 1 min intervals. Air puffs without odorant resulted in very small response amplitudes (green trace). (**B**) Representative EOG responses to benzaldehyde (1∶1000, black trace; 1:100.000, blue trace) recorded at one position on the medial surface of endoturbinate II. (**C**) Representative EOG response to benzaldehyde (1∶100.000) recorded at the medial surface of endoturbinate II; traces recorded 5 min before adiponectin application are shown in blue, those recorded 15 min after adiponectin application to that site are shown in red. Graph shows average fold-change after preincubation with adiponectin. Amplitude values before adiponectin application were set as 1. Adiponectin application leads to a 2,8-fold enhancement in EOG responses (EOG values before adiponectin application  =  1; adipo+: 2.86±0.4; P = 0.0012). Number is given as mean ± SD. (**D**) Representative EOG response to benzaldehyde (1:100.000) recorded at the medial surface of endoturbinate II; the trace recorded 5 min before application of buffer solution is shown in blue, the one recorded 15 min after buffer application to that site is shown in orange. Odorants were always applied for 500 ms. **, statistically very significant.

As a first step to scrutinize the hypothesis that adiponectin may affect the responsiveness of olfactory sensory neurons (OSNs), we have investigated the electrical response of the olfactory epithelium (OE) to odorants by recording electroolfactograms (EOG). After blowing air puffs of 500 ms containing benzaldehyde onto the medial surface of the OE, electrical responses could be recorded, as exemplarily shown in figure 1A (black curve). Air puffs without odorant resulted in only very small response amplitudes (green curve). Repeated stimulation with odorants at minute intervals typically resulted in a very similar amplitude. In order to enable a registration of changes in the response amplitude, the concentrations of odorants used were adjusted to elicit about half-maximal responses (blue curve in figure 1B). To explore, whether a pretreatment of the olfactory epithelium with adiponectin may affect the response amplitude, measurements were performed before and after a local application of a buffer solution containing adiponectin. 15 minutes after the application of adiponectin, the EOG response to an odorant application was recorded. The result of a representative experiment is shown in figure 1C. The recordings indicate that the odorant-induced EOG amplitude was clearly larger (red curve) after preincubation with adiponectin compared to pre-incubation (blue curve). The kinetic of the EOG response was not notably altered. The graph in figure 1C shows the average fold change in EOG responses (n = 3). After adiponectin application, the EOG responses were 2,8-fold increased compared to EOG responses before adiponectin application. Application of buffer solution alone did not lead to any significant change of the amplitude (figure 1D, orange curve). Thus, the results of electrophysiological recordings provided the first evidence that adiponectin can indeed change the responsiveness of the olfactory epithelium.

**Figure 2:**
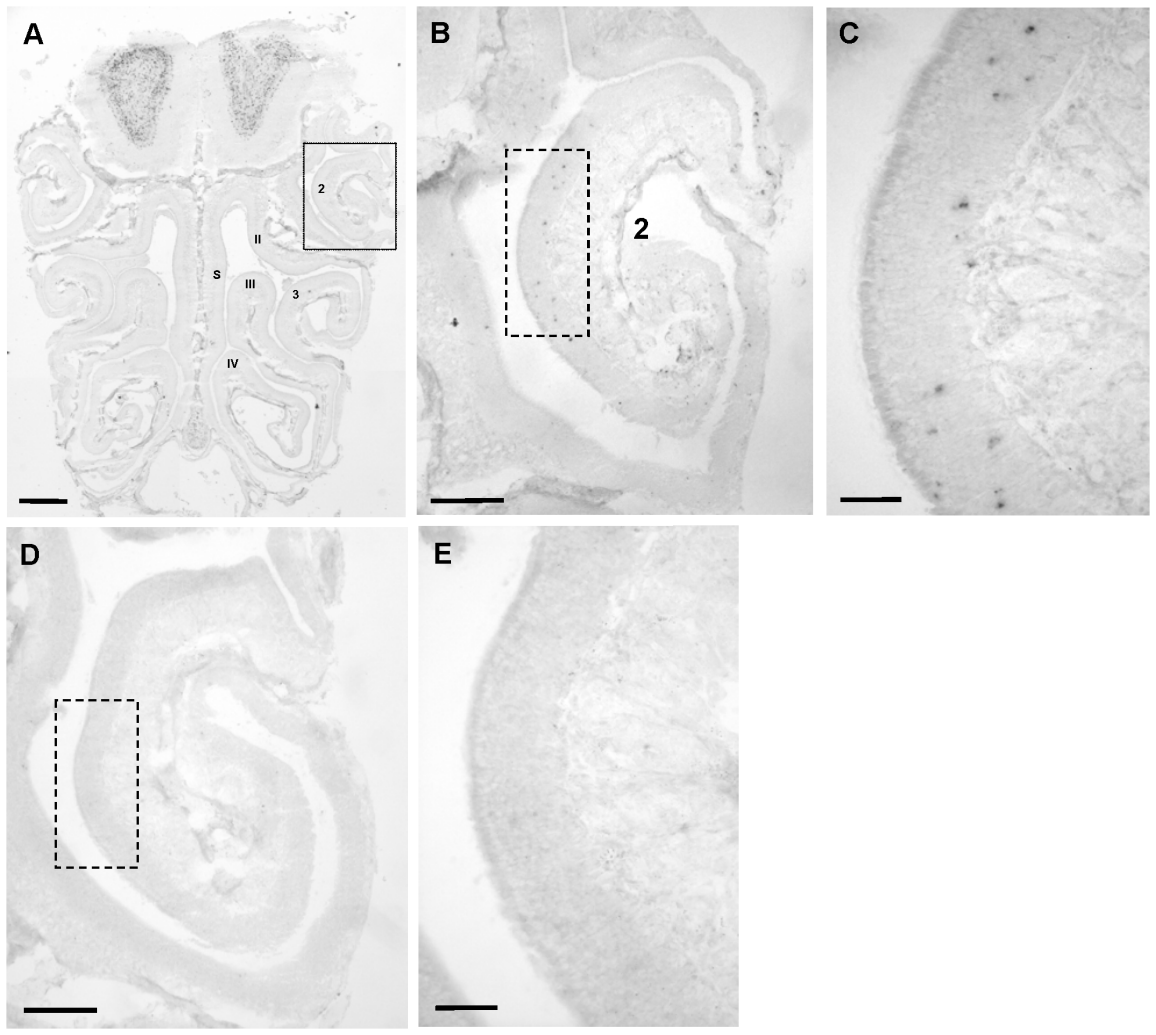
Odorant exposure elicits *Egr1*-expression in olfactory sensory neurons of the main olfactory epithelium. (**A**) Coronal section through the nasal cavity of a mouse exposed to benzaldehyde after *in situ*-hybridisation with an *Egr1*-specific antisense riboprobe. Numerals designate endoturbinates II, III, IV and ectoturbinates 2, 3; s  =  nasal septum. The box indicates the region shown in B. (**B**) Magnification showing ectoturbinate 2; the box indicates the region shown in C. (**C**) Higher magnification shows that several *Egr1*-positive cells are visible in the epithelium. (**D**) – (**E**) Without odorant exposure, hardly any *Egr-1* positive cells are visible. Scale bars: 0,5 mm in A; 0,2 mm in B, D; 50 µm in C, E.

While electroolfactogram recordings register the summed changes of electrical potentials within the epithelium induced by an odorant stimulus, it does not allow to determine what causes these changes at the cellular level; the effect could, for example be due to a stronger response of reactive cells or due to a response of more cells. Since this cannot be analyzed by studying individual cells, we set out to visualize activated OSNs within the epithelium. Towards this goal we have employed a procedure based on the upregulation of the immediate early gene *Egr1*, which has recently been used to identify activated neurons in the vomeronasal epithelium [17]. For this purpose cross sections through the nasal cavity were probed with an *Egr1* specific antisense riboprobe (figure 2A). On tissue sections from mice exposed to benzaldehyde, numerous positive cells were visible in the olfactory epithelium. A typical result is shown for a region located on the ectoturbinate 2 (figure 2B/2C). Similar results were obtained when mice were exposed to other odorants (data not shown). In contrast, on tissue sections from mice which were exposed to clean air, only few *Egr1* expressing OSNs were visible in the entire OE; on the ectoturbinate 2 hardly any *Egr1*-positive cell was visible (figure 2D/2E). These results demonstrate that monitoring the expression of *Egr1* allows to identify activated sensory neurons of the main olfactory epithelium.

**Figure 3:**
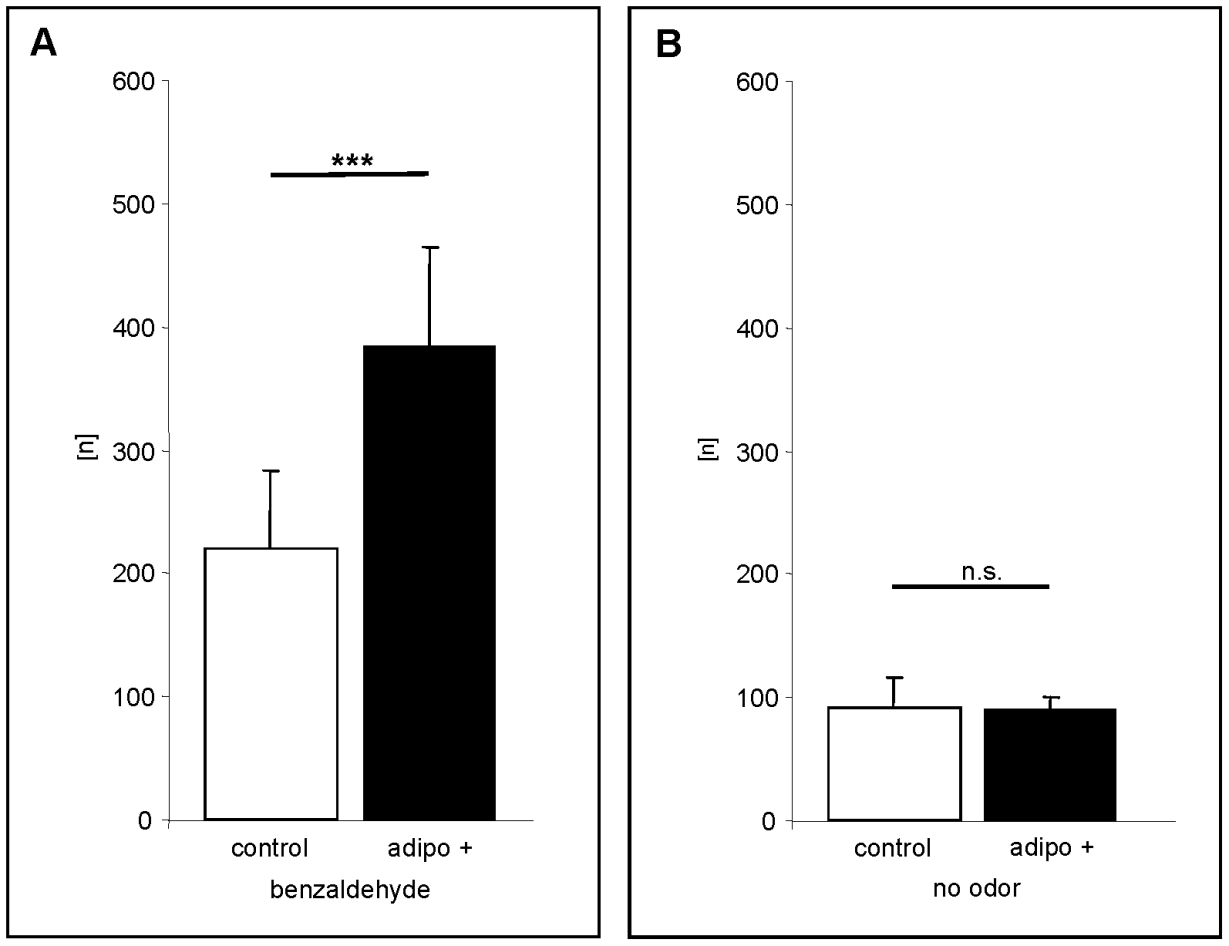
Nasal application of adiponectin causes a rise in the number of *Egr-1* positive olfactory sensory neurons after exposure to benzaldehyde. (A) Number [n] of E*gr-1* positive cells detectable on 50 consecutive sections through ectoturbinate 2 of control animals (white bar) and adiponectin-treated animals (black bar) after exposure to benzaldehyde. Adiponectin treatment significantly increased the number of labelled cells. (control: 219.6±64.5; adipo+: 383.1±80.9; P = > 0.0001) (B) Number [n] of *Egr-1* positive cells detectable on 50 consecutive sections through ectoturbinate 2 of control (white bar) and adiponectin-treated animals (black bar) without odorant exposure. Treatment with buffer or adiponectin alone did not increase the number of *Egr-1* positive OSNs. (control: 90.8±25.4; adipo+: 90.6±8.8; P = 0.9871). All numbers in this figure are given as means ± SD. ***, extremely significant; n.s. statistically not significant.

To explore the effect of adiponectin, the hormone was applied directly to the nasal epithelium of living animals; a small volume of a buffer solution containing the hormone was deposited at the nostrils of a mouse, assuming that the solution reaches the OE via the flow of mucus which moves in posterior direction towards the pharynx. This procedure has recently been successfully employed to deliver nonvolatile compounds [18] and odorant binding proteins [19] to the relevant parts of the olfactory epithelium. After the application of buffer or buffer containing adiponectin followed by an incubation period of 30 minutes, mice were exposed to benzaldehyde. Subsequently, the number of *Egr1* positive OSNs on ectoturbinate 2, which is a closely circumscribed anatomical structure, was determined. As can be seen in figure 3A, the number of labeled cells was significantly higher in adiponectin treated animals (n = 10) compared to buffer treated mice (n = 10). These data further support the idea that adiponectin indeed increases the sensitivity of olfactory sensory neurons to odorants. To exclude the possibility that the application of liquid to the nose or the application of adiponectin alone may induce *Egr1* expression, control experiments were performed. The results depicted in figure 3B indicate that a treatment of mice with buffer or with adiponectin alone (without odorant exposure) did not lead to an increased number of *Egr1* positive cells.

**Figure 4:**
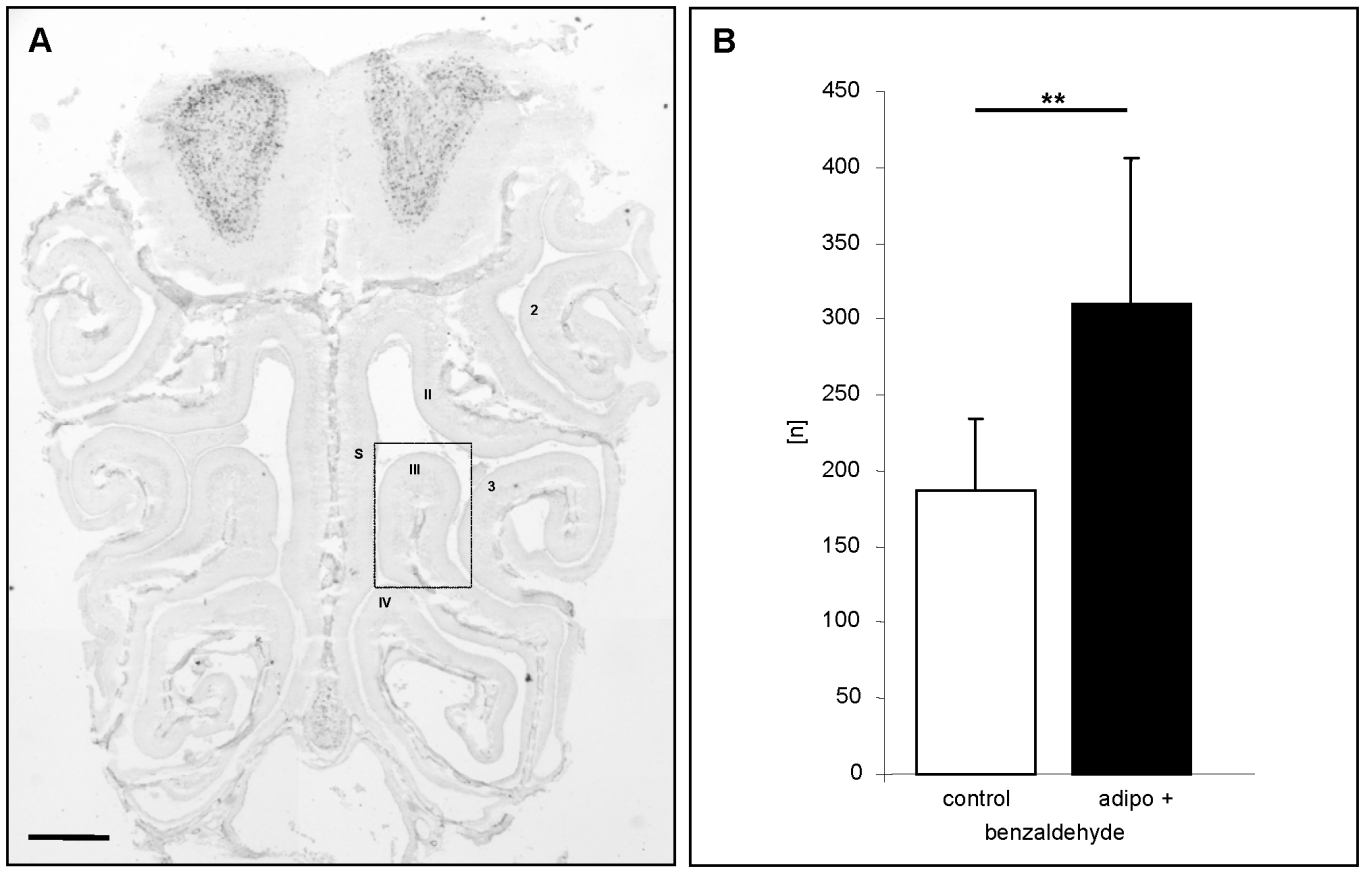
Adiponectin leads to an increased responsiveness of OSNs in different regions of the olfactory epithelium. (**A**) Coronal section through the nasal cavity of a mouse exposed to benzaldehyde after *in situ*-hybridisation with an *Egr1*-specific antisense riboprobe. Numerals designate endoturbinates II, III, IV and ectoturbinates 2, 3; s  =  nasal septum. (**B**) Number [n] of E*gr-1* positive cells on 50 consecutive sections through endoturbinate III of control animals (white bar) and adiponectin-treated animals (black bar) after exposure to benzaldehyde. The number of *Egr-1* positive cells is significantly higher when animals are pretreated with adiponectin. (control: 187.1±47.5; adipo+: 310±96.4: P = 0.0020). All numbers in this figure are given as means ± SD. **, statistically very significant. Scale bar: 0,5 mm in A.

**Figure 5:**
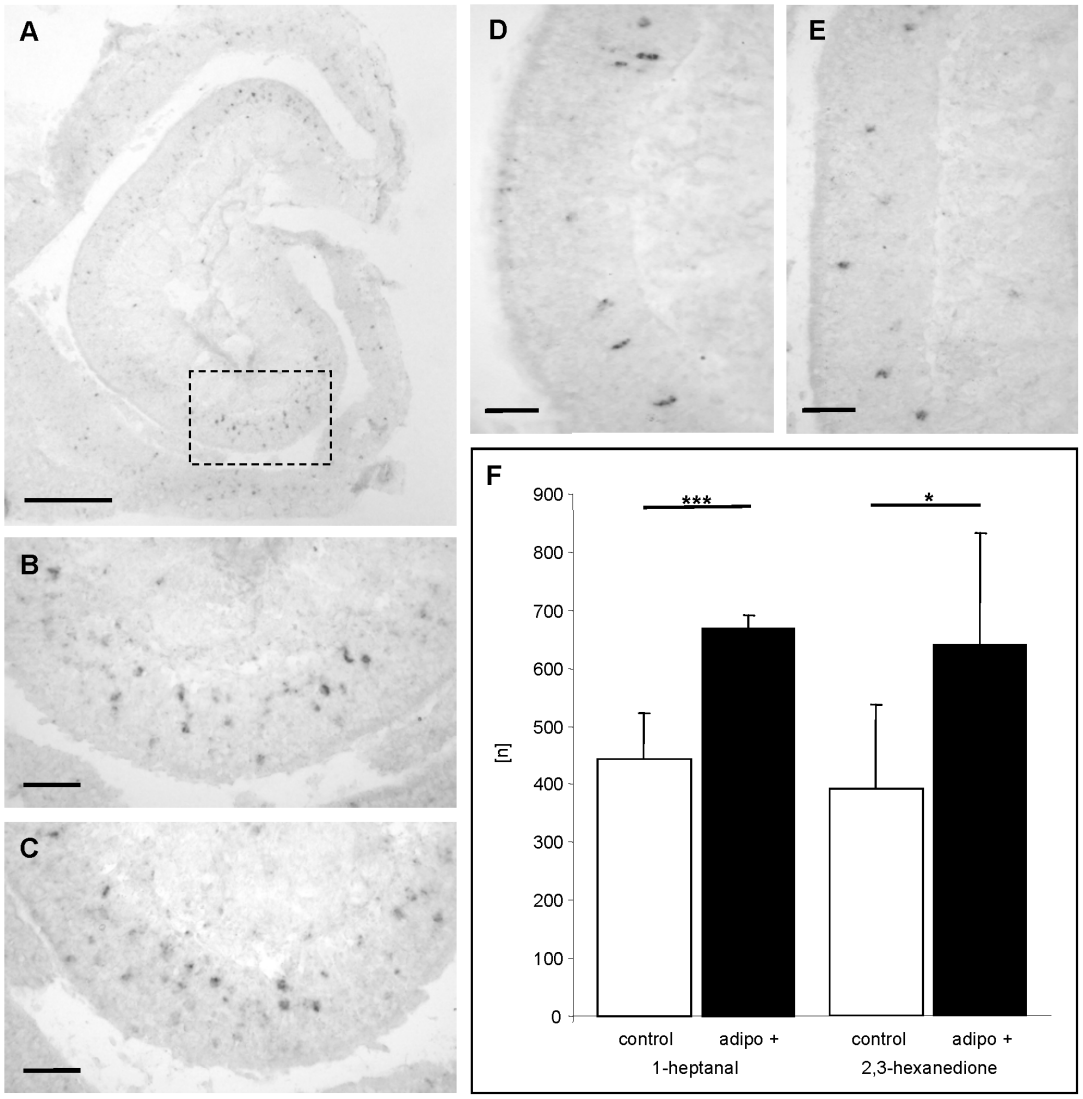
Adiponectin increases the responsiveness of OSN populations which react to different odorous compounds. (**A**) Coronal section through ectoturbinate 2 of a mouse treated with buffer solution and exposed to 1-heptanal after *Egr-1* in situ-hybridisation. The boxed area is shown in B. (**B**) High magnification of the epithelium shows numerous *Egr1* positive cells. (**C**) High magnification of the same region as in B from a mouse treated with adiponectin and exposed to 1-heptanal. Numerous OSNs are E*gr-1* positive. (**D - E**) High magnification showing an area on ectoturbinate 2 from a buffer-treated mouse (D) and an adiponectin-treated mouse (E) after exposure to 2,3-hexanedione. (**F**) Number [n] of E*gr-1* positive cells on 50 consecutive sections through ectoturbinate 2 of control (white bars) and adiponectin-treated animals (black bars). Adiponectin increases the number of *Egr-1* positive cells for both odorants. (1-heptanal: control: 442.6±80.7; adipo+: 668.2±24.4; P = 0.0003; 2,3-hexanedione: control: 390.3±145.7; adipo+: 637.8±193.2; P = 0.0311). All numbers in this figure are given as means ± SD. ***, extremely significant; *, significant. Scale bar: 0,2 mm in A; 50 µm in B-E.

Since it was previously shown that all mature olfactory sensory neurons appear to express the adipoR1 receptor [9], we next examined whether adiponectin leads to an increased sensitivity of OSNs also in other regions of the epithelium. Analyses of endoturbinate III (figure 4A) revealed that also in this region the number of activated OSNs was significantly higher in adiponectin treated mice (n = 10) as compared to buffer treated animals (n = 10) (figure 4B). To exclude the possibility that the increased responsiveness may be uniquely for benzaldehyde but rather than that the responsiveness of the olfactory epithelium was generally increased, a series of experiments was performed using the structurally quite different odorous compounds 1-heptanal and 2,3-hexanedione which most likely activate distinct OSN populations. It was found that upon exposure to 1-heptanal a variety of *Egr1* expressing cells was visible in both buffer treated and adiponectin treated mice (figure 5A-C). Interestingly, the activated cells were located in a different region of the ectoturbinate 2 than the benzaldehyde responsive cells (figure 5A). Counting the number of *Egr1* expressing OSNs revealed that after adiponectin treatment the number of 1-heptanal reactive cells was about 1.5-fold higher than control (figure 5F). A similar result was also obtained for 2,3-hexanedione (figure 5D/5E); in adiponectin treated animals the number of *Egr1* expressing cells was 1.6-fold higher (figure 5F). Together, these results indicate that adiponectin induces an increased sensitivity of olfactory sensory neurons responding to very different odorous compounds.

**Figure 6:**
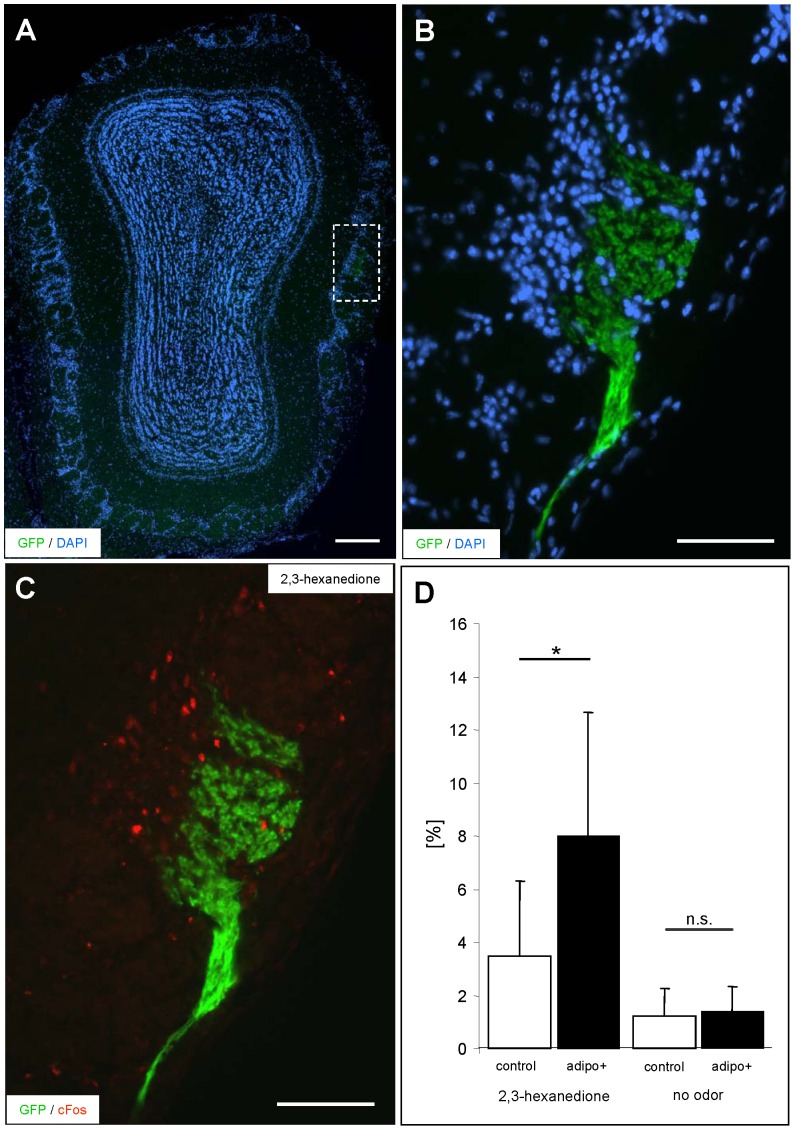
Treatment with adiponectin increases the number of *c-fos* positive juxtaglomerular cells surrounding the mOR256-17 glomeruli in mice exposed to 2,3-hexanedione. (A) Coronal section through the olfactory bulb of an mOR256-17-GFP transgenic mouse exposed to 2,3-hexanedione; the lateral mOR256-17 glomerulus (indicated by the white box) is visible by its intrinsic GFP-fluorescence. Counterstaining with DAPI. (B) Higher magnification of the mOR256-17 glomerulus shown in A. (C) Representative picture of the *c-fos* positive juxtaglomerular cells (red) surrounding the glomerulus after exposure of mice to 2,3-hexanedione. (D) Percentage [%] of *c-fos* positive juxtaglomerular cells induced by 2,3-hexanedione in mice treated with buffer solution (left white bar) or adiponectin (left black bar). In adiponectin-treated mice exposed to the odorant, a significantly higher number of *c-fos* positive cells is found at the mOR256-17 glomerulus (control: 3.5±2.7%; adipo+: 8.0±4.5%; P = 0.0226). Without odorant exposure, the number of *c-fos* positive cells is low and not significantly different in buffer-treated (right white bar) and adiponectin-treated (right black bar) mice (control: 1.2±0.15%; adipo+: 1.4±0.13; P = 0.6852). All numbers in this figure are given as means ± SD. Scale bar: 0,2 mm in A; 100 µm in B and C.

The increased number of *Egr1* expressing olfactory sensory neurons after adiponectin treatment suggests that adiponectin induces an increased sensitivity of the olfactory cells. The question, if the cells which already responded prior to the hormone treatment give a stronger response after adiponectin treatment is difficult to study directly on individual cells. However, a stronger response of a sensory neuron would imply that a stronger signal is conveyed to the glomerulus in the olfactory bulb. Thus, the response intensity of receptor-specific OSN populations would be reflected in the activity of the corresponding glomeruli. Based on the recent observation that the number of *c-fos* positive juxtaglomerular cells (JCs) correlates with the intensity of an odor stimulus [16], we have scrutinized this concept by exploiting the expression of *c-fos* in the JCs of an identified glomerulus. For this approach, we have studied the transgenic mouse line mOR256-17-IRES-tauGFP. In these mice all olfactory neurons expressing the odorant receptor mOR256-17 as well as the appropriate glomeruli in the olfactory bulb can be visualized due to their intrinsic fluorescence [11] (figure 6A-C). As a potential ligand for the mOR256-17 receptor the compound 2,3-hexanedione was recently identified in a heterologous expression system [20]. Determining the number of *c-fos* positive JCs at the periphery of the mOR256-17 glomerulus from animals which were not exposed to odorants resulted in only a very small percentage of labeled cells (data not shown). After exposure of mice to 2,3-hexanedione a significant number of *c-fos* positive cells was found (figure 6C). These results indicated that not only *in vitro* but also *in vivo* cells, which express the mOR256-17 receptor, are activated by 2,3-hexanedione. Subsequently, mice were analyzed which were treated with buffer containing adiponectin or buffer alone prior to odorant exposure. It was found that the number of labeled cells surrounding the glomerulus was significantly higher in adiponectin treated mice; the percentage of *c-fos* positive cells was almost doubled (figure 6D). Treatment with buffer or adiponectin alone did not lead to an increase of *c-fos* positive cells (figure 6D). These results indicate that pretreatment with adiponectin leads to an elevated neuronal output of distinct olfactory neurons upon odor stimulation. Together, the results strongly support the view that adiponectin increases the responsiveness of olfactory sensory neurons.

## Discussion

The discovery that mature olfactory sensory neurons in the main olfactory epithelium express the adiponectin receptor subtype adipoR1, has led to the hypothesis that the hormone adiponectin, which is considered as an important starvation signal, may affect the responsiveness of the olfactory system [9]. Using different *in vivo* approaches, in this study we demonstrate that adiponectin indeed renders the olfactory system more responsive to odorants. The results of EOG recordings have shown that under the influence of adiponectin an enhanced electrical response of the epithelium is generated upon odor stimulation (figure 1). This result is in line with the observation of a previous study demonstrating that in rats the application of neuropeptide Y leads to a stronger odorant-induced EOG response; in this case, however, only in fasted animals [21]. This obvious difference correlates with the fact, that the relevant receptor for the NPY effects is upregulated in fasted animals whereas the relevant receptor for adiponectin adipoR1 is permanently expressed in all mature OSN, independent of the feeding state.

Since an EOG recording reflects a summation of all electrical potential changes which occur in the olfactory epithelium as a response to an odor stimulus, it is not possible to easily deduce information about the underlying processes and thereby elucidate what parameter may cause the enhanced response induced by adiponectin. The hormone could either enhance the reactivity of the responding cell population or could generally increase the sensitivity of all OSNs and thereby leading to a recruitment of cell populations which normally would not respond. The results that application of adiponectin leads to an increased number of odorant-reactive OSNs (figures 3A, 4B, 5F) indicate that additionally “low-affinity receptor” cell populations were recruited. Moreover, the increased number of activated juxtaglomerular interneurons of a distinct glomerulus (figure 6D) indicates that in the normally responding neurons the reaction intensity to an odor stimulus is enhanced. Both phenomena could be due to lower response thresholds (higher sensitivity) which would turn “non-responsive” OSNs into responsive ones and would increase the reaction of “normally” responsive OSNs. Such a threshold change could either be due to a more efficient transduction process by modulating elements of the transduction cascade; alternatively, it is also conceivable that the threshold for transforming the receptor potential into action potentials is changed. This latter view would be in line with the results of previous studies indicating that adiponectin changes the excitability of magnocellular neurons of the paraventricular nucleus [22]. Furthermore, by patch clamp recording experiments Hoyda and Ferguson [8] have demonstrated that the change of excitability is mediated through a modified potassium conductance of the membrane. How this is accomplished as a consequence of an activation of the adiponectin receptor is unclear. However, in a recent study examining the effects of adiponectin in the arcuate nucleus it has been shown that activation of adipoR1 leads to a phosphorylation of the AMPK-activated protein kinase and consequently to an increased level of ATP [5]. Since many neurons comprise KATP channels, an alteration of the ATP concentration would affect the channel conductance with immediate consequences on the membrane potential and firing rate [23]. Whether such a mechanism is also involved in the adiponectin induced increase in responsiveness and/or sensitivity of olfactory neurons requires further investigation. In this context, it is also of interest where in OSNs the adiponectin receptor protein is located; unfortunately, immunohistochemical data showing its distribution are currently not available. Based on the aforementioned considerations, the receptor for adiponectin could be located in the ciliary compartment to modulate the transduction cascade. Previous studies have demonstrated that for example distinct receptors for orexins are present in this particular compartment [24]. It is also conceivable that adiponectin receptors are inserted in the membrane of the soma, close to the site where they can affect the generation of action potentials and the firing rate of the OSN. Such a localization would place them in closer vicinity to the blood vessels which pervade the *lamina propria;* adiponectin is supposed to originate from the blood stream and to diffuse from there into the mucosa. For compounds like leptin, a free diffusion through the mucosa has recently been demonstrated [25].

Since all mature olfactory sensory neurons express adipoR1 receptors, it can be assumed that adiponectin can modulate the responsiveness of all olfactory neurons, independent of which odorant receptors they express. This notion was supported by the observation that the response to structurally very different odorous compounds is increased by adiponectin. These results are in line with studies demonstrating that under fasting conditions or after systemic infusion of orexigenic peptides, the olfactory system is more reactive to many different odorants (food and non-food odors) [26]–[28]. In fact, it is conceivable that during starvation periods it would be very beneficial or even essential for survival to increase the responsiveness of the main olfactory system, since food odors are often composed of numerous odorous compounds. It is of particular interest in this context that other olfactory subsystems, notably the vomeronasal organ or the Grueneberg ganglion do not express receptors for adiponectin [9]. Both subsystems are supposed to be specialized for recognizing very unique signals, e. g. social signals, but are not involved in finding food sources.

The results that application of adiponectin leads to a higher number of activated interneurons of a distinct glomerulus (figure 6) not only indicates that the reaction of “responsive” OSNs is increased but also that stronger electrical activity is generated in the olfactory bulb which increases the probability for neuronal activity in higher brain centers. This may then lead to altered olfactory driven behavior including food searching behavior. It is thus conceivable that the starvation signal adiponectin increases the probability to find food sources which are normally not found and thereby contribute to survive periods of famine.
